# Association of glucose transporter 4 genetic Polymorphisms with obstructive sleep apnea syndrome in Han Chinese general population: a cross-section study

**DOI:** 10.1186/1476-511X-13-12

**Published:** 2014-01-12

**Authors:** Ting Yin, Nan fang Li, Mulalibieke Heizhati, Juhong Zhang, Jingjing Zhang, Ling Zhou, Guijuan Chang

**Affiliations:** 1 Institute of Hypertension of the People′s Hospital of Xinjiang Uyger Autonomous Region, Center of Diagnosis, Treatment and Research of Hypertension, Xinjiang, 91, Tianchi Road, Urumqi, Xinjiang, China

**Keywords:** Genetics, Obstructive sleep apnea syndrome, Glucose transporter 4

## Abstract

**Background:**

Obstructive sleep apnea syndrome (OSAS) is strongly associated with the increasing prevalence of cerebrovascular events and metabolic syndrome. A growing number of studies have shown OSAS is an independent factor for insulin resistance, glucose intolerance and type2 diabetes. However, relationship of OSAS with dysglycemia is complex and still remains poorly understood. Glucose transporter 4 (GLUT4) gene is Human and rodents’ main glucose transporter sensitive to insulin, and therefore confirmation of candidate gene polymorphisms and association with OSAS is needed. Aim of our study was to assess whether GLUT4 gene polymorphisms are associated with OSAS.

**Methods:**

Patients hospitalized at People’s Hospital of Xinjiang were selected from January to December 2010. A total of 568 Han subjects who possibly exist OSAS base on a history and physical examination were completed the polysomnography, 412of whom (72.5%) were diagnosed with OSAS, and 156 individuals were confirmed without OSAS (27.5%). 96 severe OSAS patients chosen from OSAS were used for DNA sequencing in functional domain. Blood samples were collected from all subjects and genotyping was performed on DNA extracted from blood cells.

**Results:**

We performed GLUT4 genome sequencing, found 4 mutated sites. And finally selected three mutated sites such as rs5415, rs4517 and rs5435, according to principle of linkage disequilibrium (*r*^*2*^ > 0.8) and minimum gene allele frequency > 5%. All SNPs satisfied HEW (*P* > 0.05). Our study demonstrated a significant association of GLUT4 SNPrs5417 allele with OSAS, compared with controls (*P* < 0.05). Haplotype H1 (TCC) and H3 (CCC) defined as SNPrs5415, rs4517 and rs5435 are marginally associated with OSAS (*P* < 0.05). Frequencies of C haplotype of rs5417 in OSAS were higher than in controls. After adjustment for confounding factors, (AC + AA) genotype significantly reduces prevalence of OSAS, compared with CC genotype. Level of awake blood oxygen and lowest blood oxygen of (AA + AC) genotype was significantly superior to those of CC genotype.

**Conclusions:**

Our study demonstrates GLUT4 gene SNPrs5417 is associated with OSAS in hypertensive population. Carriers of AA + AC have less prevalence of obstructive sleep apnea syndrome than that of CC carriers.

## Background

OSAS is defined as coexistence of excessive daytime sleepiness with irregular breathing at night due to complete or partial closure of upper airways [1,2]. OSAS is estimated to affect 2-4% of adult population worldwide [3]. All components of Metabolic syndrome, including hypertension [4,5], insulin resistance(IR) [6,7], and low HDL-C, together with high levels of TG [8,9] are frequent in patients with OSAS. In this context, OSAS was considered a “manifestation of MetS” [10]. Recently, American academy of Sleep Medicine (AASM) in "Sleep2013: the united association of professional sleep the 27th annual meeting", recommended all individuals with type 2 diabetes mellitus and hypertension evaluate their have sleep conditions [11]. However, relationship of OSAS with dysglycemia is unclear. There have been accumulating evidences suggesting OSAS and IR are independently associated [12,13], considering mechanisms such as activations of sympathetic nervous system, hypothalamic-pituitary axis, sleep fragmentation and changes in inflammatory pathways in OSAS [14,15]. GLUT4 is expressed most prominently in adipocytes, skeletal muscles, and myocardiocytes, where it functions as so-called insulin-responsive glucose transporter [16]. Closely associated with insulin resistance and type 2 diabetes mellitus(DM), decrease in GLUT4 expression in cells and inhibition of its transposition are important factors causing dysglycemia [17,18]. Muscle contraction and hypoxia are believed to activate glucose transportation by a calcium-mediated mechanism [19,20]. However, so far, progress in determining genotypes of OSAS is affected by lack of a consistent definition of phenotype [21], especially at the part of dysglycemia. This study hypothesized indirectly, from molecular genetic mechanisms that OSAS causes dysglycemia phenotype, that OSAS might be associated with GLUT4 gene polymorphism through GLUT4 gene mutation locus from sequencing screening. We aim to provide new ideas for control of risk factors for cardiovascular diseases from molecular genetics aspects.

## Results

### GLUT4 genetic sequence and based typing

Our study found four locus in GLUT4 gene of 96 subjects with severe OSAS, according to linkage disequilibrium(LD) (*r*^*2*^ > 0.8) and minimum gene allele frequency > 5%. Representative locus are SNPrs5415, rs5417 and rs5435 (Shown in Table 1).

**Table 1:** GLUT4 genetic sequence and based typing

**SNPs**	**LD**	**Region**	**Allele 1 freq**	**Allele 2 freq**	**The change of bases and flank sequence of variable sites**	**dbSNP ID**
573C/T		promoter	0.72	0.23	TGTCGCGGAC[C/T]CTTTAAGGCG	rs5415
9A/C	a	Exon 1	0.35	0.65	CCCGCTCCAC[A/C]AGATCCGCGG	rs5417
A/G	a	Exon 1	0.35	0.65	TGCTCTCCGG[A/G]TCCTTGGCTT	rs5418
2070C/T		Exon 4	0.64	0.36	GCCTGGCCAA[C/T]GCTGCTGCCT	rs5435

### Characteristics of recruited subjects

Case group included 412 hypertensive subjects with OSAS, mean age (46.31 ± 9.99), gender distribution (80.8% male); control group encompassed 156 hypertensive subjects without OSAS, mean age (42.15 ± 8.81), gender distribution (66.7% male). There is statistically significant difference in age, BMI, neck circumference, abdominal circumference, Hypersensitive c-reactive protein, fasting glucose, triglycerides, total cholesterol /high-density lipoprotein cholesterol, apnea hypopnea index (AHI), awakening respiratory index, index of periodic leg movement, average heart rate, average duration of obstructive apneas, average blood oxygen saturation and minimal blood oxygen saturation, and frequency of Oxygen saturation decreasing more than 5% or higher between two groups (*P* <0.05) (Shown in Table 2).

**Table 2:** Characteristics of recruited subjects

	**Control group**	**Case group**	***P***
Subjects n	156	412	-
Sex ratio M:F	104/52	333/79	<0.001
Age yrs	42.15 ± 8.81	46.31 ± 9.99	<0.001
SBP mmHg	144.02 ± 15.67	146.04 ± 16.16	0.231
DBP mmHg	99.52 ± 10.29	99.76 ± 11.54	0.838
BMI kg/m^2^	26.22 ± 3.45	28.24 ± 3.53	<0.001
Neck circumferencecm	38.17 ± 5.22	40.99 ± 4.20	0.030
abdomen circumference cm	93.21 ± 9.69	99.75 ± 9.23	<0.001
hemoglobin	146.80 ± 15.42	148.42 ± 14.83	0.307
hs-CRP	1.45 ± 1.94	2.45 ± 3.94	0.008
FPG	5.02 ± 0.89	5.39 ± 1.47	0.010
total carbon dioxide	24.32 ± 2.63	24.90 ± 2.61	0.038
CHOL	4.40 ± 0.72	4.56 ± 0.88	0.079
TG	1.87 ± 0.47	2.35 ± 0.64	0.002
LDL-C	2.49 ± 0.61	2.54 ± 0.69	0.446
HDL-C	1.18 ± 0.32	1.11 ± 0.30	0.012
CHOL/HDL-C	3.96 ± 1.06	4.36 ± 1.27	0.001

hs-CRP: high-sensitivity C-reactive protein; FPG: fasting peripheral glucose; CHOL: total cholesterol; TG: triglyceride; LDL-C: low-density lipoprotein cholesterol ;HDL-C: high-density lipoprotein cholesterol.

### Comparison of glucose from OGTT and insulin releasing between two groups

**Figure 1:**
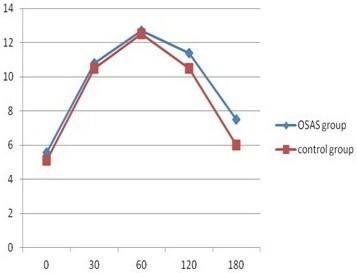
The level of OGTT between case-control group.

**Figure 2:**
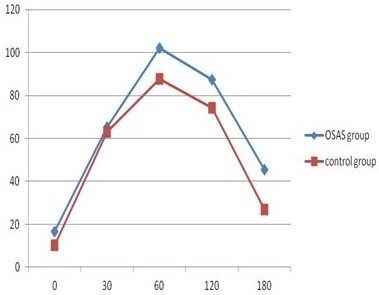
The level of insulin release test between case-control group.

**Figure 3:**
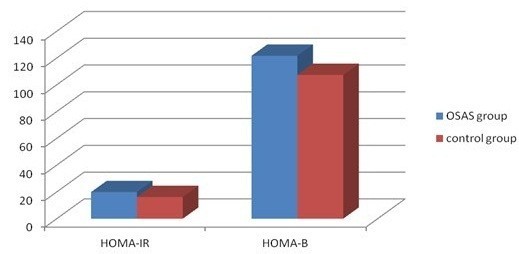
Compare of HOMA-IR and HOME-B Index between case-control group.

Of 568 subjects, 374 were confirmed to have no DM, whereas 194 subjects had FPG >5.6 mmol/L after fasting 12 h, to 164 of whom OGTT and insulin releasing test were conducted according to their acceptance. Figure 1 presents oral glucose tolerance curve, and statistical significance was found to exist in FPG of two groups (5.35 ± 1.4 > 5.1 ± 1.0,P = 0.012); FPG of OSAS group was higher than that of control group. Although OGTT was taken at four time points (30 min, 60 min, 120 min, 180 min), no statistical significance was found between two groups (*P* > 0.05); however curve contained tendency to be higher than that of control group after OGTT of OSAS group, particularly 2 h after OGTT. Figure 2 reveals curves of insulin releasing tests of two groups; fasting insulin in OSAS group was higher than in control group (16.5 ± 7.8 > 12.3 ± 7.3, *P* = 0.007). However, statistical significance was not found in insulin levels of two groups after 30 min, 60 min, 120 min, 180 min of OGTT (*P* > 0.05). According to OGTT-IRT, associated indicators for evaluation of insulin resistance and B cell insulin releasing capacity include HOMA-IR, Insulin secretion Index (HOME-B),whereas differences in HOMA-IR and HOME-B of two groups were not found to exist statistical significance (*P* > 0.05) (Show in Figure 3).

### SNPs in *GLUT4* are associated with OSAS

Before association analysis, all SNPs were subjected to Hardy Weinberg Equilibrium(HWE) which suggested that genotypes of all SNPs were in HWE proportions and none of the SNPs was deviated from HWE. Total number of subjects is 568, with 11 genotyping failure. Difference between SNPrs5417genotype frequency and allele frequency distribution was statistically significant between case group (405 cases) and control group (152 cases). Statistic difference was found between gene frequency and allele frequency and GLUT4gene SNPrs5417 in males after gender-stratified analysis (P < 0.05), but the difference does not exist in female subjects. No association was found between rs5415 and rs5435 locus of GLUT4 gene and OSAS (P > 0.05) (Shown in Table 3).

**Table 3:** Genotype and allele distributions for three polymorphisms of GLUT4 in control and in patients with OSAS

**Polymorphisms**		**Total**		**Male**		**Female**	
	**Genotypes**	**Control n (%)**	**OSAS group n (%)**	**Control n (%)**	**OSAS group n (%)**	**Control n (%)**	**OSAS group n (%)**
rs5417	AA	28 (18.4)	53 (13.1)	22 (22.0)	46 (14.1)	6 (11.5)	7 (9.0)
	AC	85 (55.9)	200 (29.4)	56 (56.0)	151 (46.2)	29 (55.8)	49 (62.8)
	CC	39 (25.7)	152 (37.5)	22 (22.0)	130 (39.8)	17 (32.7)	22 (28.2)
			Χ^2^ = 7.630, *P* = 0.022		Χ^2^ = 11.332,*P* = 0.003		Χ^2^ = 0.673, *P* = 0.714
	A	141 (46.4)	306 (53.6)	100 (50.0)	243 (37.2)	41 (39.4)	63 (40.4)
	C	163 (53.6)	504 (62.2)	100 (50.0)	411 (62.8)	63 (60.6)	93 (59.6)
			Χ^2^ = 6.811, *P* = 0.009		Χ^2^ = 10.514, P = 0.001		Χ^2^ = 0.024, *P* = 0.877
	CC	73 (48.0)	209 (51.4)	54 (52.9)	170 (51.7)	19 (38.0)	39 (50.0)
	CT	67 (44.1)	154 (37.8)	40 (39.2)	120 (36.5)	27 (54.0)	34 (43.6)
	TT	12 (7.9)	44 (10.8)	8 (7.8)	39 (11.9)	4 (8.0)	5 (6.4)
rs5415			Χ^2^ = 2.272, *P* = 0.321		Χ^2^ = 1.330,*P* = 0.514		Χ^2^ = 0.668, *P* = 0.716
	C	213 (70.1)	572 (70.3)	148 (72.5)	460 (69.9)	65 (65.0)	112 (71.8)
	T	91 (29.9)	242 (29.7)	56 (27.5)	198 (30.1)	35 (35.0)	44 (28.2)
			Χ^2^ = 0.004, *P* = 0.947		Χ^2^ = 0.522,*P* = 0.470	Χ^2^ = 1.319, *P* = 0.251	
	CC	66 (43.7)	187 (46.4)	44 (44.4)	157 (47.9)	22 (42.3)	30 (40.0)
	CT	71 (47.0)	169 (41.9)	48 (48.5)	128 (39.0)	23 (44.2)	41 (54.7)
	TT	14 (9.3)	47 (11.7)	7 (7.1)	43 (13.1)	7 (13.5)	4 (5.3)
rs5435			Χ^2^ = 1.400, *P* = 0.497		Χ^2^ = 4.209, P = 0.122		Χ^2^ = 3.046 *P* = 0.218
	C	203 (67.2)	543 (67.4)	136 (68.7)	542 (71.7)	67 (64.4)	101 (67.3)
	T	99 (32.8)	263 (32.6)	62 (31.1)	214 (28.3)	37 (35.6)	49 (32.7)
			Χ^2^ = 0.002, *P* = 0.962		Χ^2^ = 4.690, *P* = 0.406	Χ^2^ = 0.232, *P* = 0.630	

### Haploid type of GILU4 gene polymorphism locus in two groups

SNP analyzer software was used to analyze Matching linkage disequilibrium analysis and haplotype separate analysis of three locus of GLUT4 gene, and D′ was found to be 0.9, suggesting rs5417, rs5415 and rs5435 locus were in incomplete linkage disequilibrium in our study subjects, which needs further separate analysis. Most frequent variable sites, SNP r5415, rs5417 and rs5435, can constitute 6 haplotypes, of which frequencies > 4% are 4 types. Most common haplotypes account for 96.64% of haplotypes in OSAS patients, and statistically significant difference was found in distribution of H1 (rs5415T-rs5417C-rs5435C) and H3 (rs5415C-rs5417C- rs5435C) in two groups (P < 0.05), and frequencies of C haplotype in case group were higher than in controls (shown in Table 4).

**Table 4:** Haplotype distributions for three polymorphisms of GLUT4 in control and in patients with OSAS

	**Haplotype**	**Overall (%)**	**case (%)**	**control (%)**	***P***
H1	T-C-C	38.28	36.28	4.37	0.025
H2	T-C-T	27.37	2.71	28.09	0.806
H3	C-C-C	26.75	28.58	21.77	0.024
H4	C-C-T	4.24	4.65	3.2	0.299

### Adjustment for confounding factors

After adjusting such confounding factors of OSAS as gender, age, body mass index (BMI) hemorrheologic and sleep parameters using logistic regression, (AA + AC) genotype was still an independent protective factor for OSAS individuals (*OR*=0.362, *95%CI* 0.159~0.823, *P* = 0.015). Additionally male, more than 50 years, overweight, obesity and lowest arterial oxygen saturation were significant predictive factors associated with OSAS individuals comparing with controls (shown in Table 5).

**Table 5:** Logstic regression correct the confounding factors of OSAS

**Variable**	**Coefficient of regression**	**Standard error**	**Wald**	**DOF**	***P***	***OR *** **Value**	**95.0% *C.I. *** **for *OR***	
							**Lower**	**Upper**
Tender(male)	0.836	0.238	12.384	1	0.000	2.307	1.448	3.676
Age(>50)years	0.796	0.245	10.568	1	0.001	2.217	1.372	3.582
BMI ≥ 24Kg/m^2^	1.022	0.323	10.022	1	0.002	2.779	1.476	5.234
Lowest arterial oxygen saturation	0.341	0.069	25.256	1	0.000	1.406	1.231	1.605
rs5417(AA + AC)	−1.016	0.419	5.881	1	0.015	0.362	0.159	0.823

### Association analysis of *GLUT4* rs5417 with OSAS-related biochemical parameters

One-way association analysis between the mutated locus and OSAS-related biochemical parameters and PSG-related parameters found awake oxygen saturation and minimal oxygen saturation in (AA + AC) genotype superior to those in CC genotype in OSAS males of Han population (P < 0.05), whereas average heart rate and systolic blood pressure in (AA + AC) genotype were lower than in CC genotype (P < 0.05) (shown in Table 6).

**Table 6:** Comparison of sleep parameters phenotype among different genotypes of SNP in GLUT4 gene

**rs5417 Genotype**	**awake blood oxygen**	**lowest blood oxygen**
AA + AC	91.65	75.71
CC	90.61	73.29
*P*-value	0.007	0.039
*P*-value#	0.014	<0.001
*OR*	2.274	1.716
*Beta values*	0.823	0.540

Data are presented as mean ± SD unless otherwise stated. *P* values# and beta values were adjusted simultaneously for age, gender and BMI by a general linear regression.

## Discussion

Our study first reports GLUT4 gene SNPrs5417 allele is significantly associated with diagnosis of OSAS in hypertensive population. In addition, significant association is found between GLUT4 gene SNPrs5417 and males with OSAS. A allelic frequency in case group is lower than in control group. Significant relation is found between GLUT4 gene and males with OSAS in Han population, whereas the relation is not found in female subjects.

It is unanimously recognized that OSAS has familial aggregation and genetic susceptibility; however its genetic mechanism remains unknown so far. More than 72% in our study subjects have hypertension coupled with OSAS, and other epidemiological studies have indicated that prevalence of OSAS in hypertensive population is up to 50% ~ 60% [22]. Hypertension and OSAS often coexist, and our previous study found patients merging with OSAS and hypertension are more likely to have metabolic disorders [23,24]. These people are more likely to coexist insulin resistance, increased triglyceride and uric acid levels and decreased high density lipoprotein cholesterol. This study eliminated confounding factors of hypertension in OSAS patients by case–control design of particular subjects. In OSA, cycles of hypoxia and re-oxygenation facilitate the formation of reactive oxygen species (ROS) which impair endothelial function [25] and promote lipid and glucose peroxidation [26,27] and increased formation of ROS can activate inflammatory responses [28]. One of the major sources of cellular reactive oxygen species (ROS) is mitochondria, dysfunction of which contributes to several pathological conditions including vascular complications of diabetes, neurodegenerative diseases, and cellular senescence. Shorter intermittent hypoxic exposures as pathophysiology of OSAS might preferentially activate inflammatory pathways and over-expression of such inflammatory factors as TNF-α, IL-6 and IL-8 [29], which influences normal insulin signaling of adipocytes and GLUT4, bringing about reduction of glucose assimilation triggering metabolic dysregulation [30]. Furthermore, Insulin-stimulated GLUT4 translocation and glucose uptake are associated with NF- KB activation [31] which, a susceptible gene for OSAS [32] and nuclear receptor, plays a central role in inflammatory response and orchestrates over-expression of a range of factors including cytokines (TNF-a, IL-6 and IL-8) and adhesion molecules (intercellular adhesion molecule-1) [33,34]. Overwhelming findings emphasize we gain full understanding about relation between OSAS and dysglycemia. In particular it is necessary to understand hereditary susceptibility of OSA and glucose metabolism. According to analysis of baseline data in this study, concentrations of FPG and hypersensitive C-reactive protein are higher in case group than in controls, indicating coupling reactions of dysimmunity caused by GLUT4 gene-related dysglycemia such as airway inflammatory cell invasion and high airway resistance leading to hypopnea and apnea during sleep.

Sushmita [35] reported prevalence of OSAS in T2DM is around 71%. IR acts as important role of pathogenesis of T2DM [36]. Recently, total serum cholesterol/high-density lipoprotein cholesterol as evaluation index of IR has become a hotspot in cardiovascular diseases [37]. Present study found significant difference of insulin resistance in OSAS patients comparing with controls using assessment indicators. FPG and fasting insulin of OSAS group was found to be higher than those of non-OSAS group after OGTT and insulin releasing test, explaining risk for impaired glucose tolerance of OSAS patients was higher, impaired fasting glucose reflecting basal insulin level secreted from β-cell is affected, and Punjabi [38] showed that severe OSAS is associated with function of pancreatic B cells. The two indicate intermittent hypoxia and re-oxygenation during OSAS bring about elevation of ROS. β cell is sensitive to ROS which generates dysfunction of β cell and even apoptosis. Therefore we can assume patients with OSAS experience a peak of ROS after nocturnal hypoxia, which affects β cell significantly, whereas intermittent hypoxia hardly exist daytime; thus there is no difference in dawn FPG and insulin levels of OSAS and non OSAS group. This is also the reason why dawn blood pressure is monitored in order to evaluate responsiveness of CPAP therapy; meanwhile this can explain why no significant differences are found in FPG and insulin levels of OSAS and non OSAS groups after activity and oxygen intake increasing. However β-cell damage increases with progress and severity of OSAS, and type 2 diabetes mellitus develops finally.

GLUT4 is an insulin-dependent glucose transporter protein, playing a pivotal part on IR by insulin signaling pathway [39]. P38MAPK signaling pathway may improve insulin sensitivity through increasing intrinsic activity of GLUT4 and consequently increase glucose transportation and assimilation [40,41]. Lu [42] ect found relative of p-p38MAPK and GLUT4 gene expression increased in IR rats of animal models. The latest study revealed P38MAPK is sensitive to ROS [43]. Reduction of P38MAPK activity reduces tolerance of brain to anoxia [44]. Intermittent hypoxia, the hallmark of OSAS, is implicated to promote formation of ROS and to induce oxidative stress, and brings about metabolic disorders such as hypertension, T2DM, insulin resistance, which aggregates OSAS. A part from inhibiting P38 MAPK, hypoxia also inhibits Akt-mTOR in differentiating myoblasts [45]. Rena′s [46] research confirmed genetic and pharmacological evidence suggesting that suppression of Akt and mTOR activity is responsible for the loss of the myogenic action of insulinlike growth factors under hypoxia. Some other evidences indicate that selective suppression of PI3K/Akt /mTOR signaling pathway supresses inflammatory reaction caused by oxidative stress [47]. Except for this, reduction of antioxidant enzymes in pancreas β cell is both an important target spot of ROS [48], and a direct factor of OSAS to generate IR. An observational cohort study found remarkable improvement of fasting and postprandial glucose in moderate to severe OSAS patients without obesity after CPAP therapy [49]. Thus we can infer chronic oxidative stress secondary to OSAS and excessive reactive oxygen species influence activation of correlative signaling pathway and nuclear receptor to lead to abnormal activation and expression of GLUT4 gene. Treatment of OSAS contributes to regulate activity of reflective signaling pathway, increase activation of GLUT4 molecule, improve IR in OSAS patients, and decrease cardiovascular events.

Moreover, SNPrs5417 subtype of GLUT4 gene related to OSAS expressed significant difference in gender; C allelic of male case group was significantly higher than that of male control group, whereas this phenomenon does not exist in female subjects. Some animal experiments reported that melting GLUT4 of musculi soleus in male rats causes reduction of ATP and phosphocreatine, which is more obvious than those of female rats [50]. GLUT4 mainly serves as its glucose transporter in skeletal muscle and liver, difference of which in males and females may bring about different expression in the same environment after influenced by intermittent hypoxia. However, a recent Turkish study reported OSAS-induced inflammation is more obvious in females [51]. Prevalence of OSAS is dominant in males; clinical studies have been focused on males, which has lead to lack of data on females. This study did not find any relation between three locus’ mutation of GLUT4 and OSAS in female subjects, which may have some connections with sample size and racial difference. This aspect needs further molecular researches and longitudinal, large-sampled, population-based studies to explore. AA and AC types of SNP rs5417 were found to be independent protective factors for OSAS susceptibility, compared with CC type after adjustments for confounding factors using logistic regression analysis. Awake oxygen saturation and minimal oxygen saturation are higher in carriers of (AA + AC) types of GLUT4 rs5417 than in CC Homozygote types in male subjects with OSAS of Han population, which is consistent with findings from other studies. This result explains anoxia in C allelic carriers is more severe; although there is no difference in AHI of recessive genetic model, severity of OSAS in CC type carriers can be inferred. Since OSAS is a complex disease caused by genetic and environmental interaction, single mutation locus of GLUT4 is not sufficient to bring about intermediate phenotype and Phenotype change of OSAS. No association has been found between rs5415 and rs5435 gene types and OSAS, whereas haplotypes of combinations of rs5417, rs5415 and rs5435 reported to be associated with OSAS. Nair [52] ect found combined haplotypes of rs5417 and rs5418 of GLUT4 gene and combined haplotypes of rs5435 and rs5415 associated with T2DM. It is obvious that combination of GLUT4 gene and TaqSNPs increases genetic susceptibilities of OSAS and its related clinical co-morbidities.

## Conclusions

To take aforementioned evidences together, SNPrs5417 of GLUT4 gene is correlated to OSAS; Awake oxygen saturation and minimal oxygen saturation in AA homozygote of GLUT4 SNPrs5417 in Han population are higher than in CC homozygote. Elaborate regulations of cellular pathways related to GLUT4 and OSAS needs further explorations, and extension of GLUT4 genes needs further determination of relations of genetic polymorphisms and OSAS.

## Methods and subjects

### Subjects

Patients hospitalized in the center of hypertension diagnosis and treatment of People’s Hospital of Xinjiang Uygur Autonomous Region from January to December 2010 were selected, based on following exclusive and inclusive criteria. Exclusive criteria: subjects with a history of long-term alcohol-intake, asthma, bronchiectasia, severe maxillofacial deformities, thyroid diseases, chronic obstructive pulmonary diseases, acute phase of infection, history of upper-airway surgery, history of pancreas surgery, pheochromocytoma, primary hyperaldosteronism, established T2DM or taking hypoglycaemic drugs, cerebro-cardiovascular diseases within 6 months, severe hetapic and/or renal diseases and cancer were excluded from this study. Inclusive criteria: patients who had nocturnal snoring, apneas, diurnal sleepiness during reading, watching TV, or meetings lasting at least an hour and after the meal (without drinking) via medical history and physical examination; hypertensive patients with unexplained lip and/or tongue dryness, unexplained cyanosis of lip and/or nail bed, elevation of hemoglobin levels and/or hematocrit. A total of 568 Han Chinese with no miscegenation satisfied above inclusive criteria and underwent polysomnography (PSG), 412 (133 mild OSAS, 141moderate OSAS and 138 severe OSAS) of whom were diagnosed with OSAS and selected as case group on the basis of apnea-hypopnea index (AHI ≥ 5); 156 of 568 were determined to have no OSAS according to apnea-hypopnea index (AHI < 5) and selected as control group. Study protocol was approved by Ethic’s Committees of Health Ministry in Xinjiang Uygur Autonomous Region, and People’s Hospital of Xinjiang Uygur Autonomous Region; informed consent was signed by all subjects.

### Data collection

Baseline data of subjects were collected and completed immediately after their hospitalization by physicians. Questionnaire contents included age, gender, height, weight, waist circumference, heart rate, blood pressure and other general information, diagnosis or treatment history of hypertension, hypercholesterolemia, and DM. BMI was calculated by weight in Kg divided by height in squared meter. Fasting blood samples were collected from median cubital veins of each subjects after overnight PSG at sleep monitoring center in order to be used for analysis of liver and renal function, FPG, serum and urinary electrolytes, and blood lipids.

### PSG

Polysomnography (PSG) was recorded using (computer E system Australian). Sleep studies were performed in a dedicated, quiet, dark room. Nocturnal sleep duration was at least 7 hours for monitoring. No sleep deprivation, caffeine, sedation or alcohol was used. Monitoring Parameters included electroencephalography (EEG), electro-oculography (EOG) and electromyography (EMG), EEG was recorded from two scalp sites (Cz/Pz). Frontal EEG was also recorded using “mixed” channels comprising EEG/EOG signals (Cz/Fp1, Cz/Fp2). EOG was recorded from electrodes placed at sites on the outer canthus of the eye. Submental EMG was recorded using two electrodes placed on the belly of the genioglossus. A grounding electrode was placed at Fpz. Electrodes placed on the right and left anterior tibialis recorded leg EMG. Nasal oxygen catheter was used for recording oro-nasal airflow, body position and chest wall movement by electrical impedance, heart rate using an electrocardiogram and finger pulse oximetry. All signals were recorded on computer. Data were analysed by a specialized researcher (Yingchun Wang) after blinding of data using AASM scoring manual. Standard criteria [53]: ①apneas:complete oro-nasal apneas continuing more than 10s. ②hypopneas:air flow decreasing more than 50% and oxygen saturation more than 4% from baseline values, continuing over 10 seconds. ③apnea-hypopnea index, AHI = (apnea events + hypopnea events)/h.

### GLUT4 gene sequencing and polymorphism detection

We sequenced all exons and the promoter region of GLUT4 gene. Blood samples were obtained from 96(male 84,female 12) severe OSAS cases, who were chosen from OSAS group of the study population. Reagent-Kit was used to extract DNA from blood samples using phenol-chloroform extraction (PAXgene blood DNA Kit, A Q IAGEN /BD Company). Total region of exons with their flanking sequences and approximately 1.0 kb of the upstream region had PCR amplification and sequencing analysis (ABI 3130x,lB igDye Term inator Cycle sequencing V311 /V111 K it, Applied Biosystems, Foster City, Californ ia, USA). The designable set of common SNPs (minor allele frequency >5%) were genotyped in common variable sites of Han Chinese population through Sequencing. Representative mutated loci was selected according to linkage disequilibrium (LD) (r^2^ > 0.8). TaqMan- PCR (ABI PRISM7900HT Sequence Detection System, Warrington, UK) was used to determine genotypes. Reaction system of TaqMan-PCR: Master Mix 2.25 uL, Primer 0.125 uL, DNA 1.0 uL, plus deionized water until 5uL.Reaction condition of TaqMan-PCR:Initial denaturation 95°C 10 min, 43 cycle (degeneration 95°C15 s, annealing 60°C1 min, extension 71°C 1 min), extension 72°C 7 min. Quality control of genotypes was as following: blank and positive comparison was set for every 384, and case samples were randomly distributed. Read ratio Of genotypes is > 98%; concordance rate of duplicate detectionis is 100%. TaqMan PCR genotyping probe of mutation loci and primer information reported at database were acquired from US biological systems web site (http://appliedbiosystems.com).

### Statistical analysis

Before carrying out tests for association, all SNPs were tested for Hardy-Weinberg Equilibrium (HWE). SNP analyzer software was used for testing for linkage disequilibrium, haplotype analysis and genotype frequency calculation. Computer package IBM SPSS Statistical version 19 was used for statistical analysis. Data were presented as proportions, means (±SD), geometric means (SD range), or, in the case of variables that did not conform to a normal or log-normal distribution, medians (interquartile range). For independent samples, proportions by Fisher’s exact tests were used for two-way comparison, for normally distributed variables independent sample *t* test was used, and for nonnormally distributed variables, Mann–Whitney *U* test was used. Tests for association were normally done by Chi-square test for testing difference in gene frequency and allele frequency. Logistic regression analysis was used to determine whether plausible mediating or confounding variables were independently associated with outcome. Tests for association of genotypes with biochemical parameters and parameters related to sleep were done by analysis of One-way ANOVA. All p-values were for two-tailed tests and considered to be significant at a 0.05 level.

## Abbreviations

OSAS: Obstructive sleep apnea syndrome; GLUT4: Glucose transporter 4; PSG: Polysomnography; HDL-C: High-density lipoproteincholesterol; TG: Triglyceride; FPG: Fasting plasma glucose; MetS: Metabolic syndrome; IR: Insulin resistance; COPD: Chronic obstructive pulmonary diseases; SBP: Systolic blood pressure; DBP: Diastolic blood pressure; AHI: Apnea-hypopnea index; BMI: Body mass index; EEG: Electroencephalography; EOG: Electro-oculography; EMG: Electromyography; LD: Linkage disequilibrium; HWE: Hardy-weinberg equilibrium; OGTT: Oral glucose tolerance test; ROS: Reactive oxygen species; TNF-a: Tumor necrosis factor alpha; IL-6: Interleukin-6; T2DM: type 2 diabetes mellitus.

## Competing interests

The authors declared no conflicts of interest with respect to the research, authorship, and/or publication of this article.

## Authors’ contributions

NFL PHD came up with hypothesis and study design. JuZ performed laboratory analyses. JiZ and GC performed statistical analyses. MH offered help in language. All authors read and approved the final manuscript.
